# Characterization of CPCA-induced action on isolated rat femoral artery

**DOI:** 10.1186/1471-2210-11-S2-A29

**Published:** 2011-09-05

**Authors:** Miroslav Radenković, Radmila Stevanović, Milica Stojiljković, Mirko Topalović, Marko Stojanović

**Affiliations:** 1 Department of Pharmacology, Clinical Pharmacology and Toxicology, Medical Faculty, University of Belgrade, 11129 Belgrade, Serbia; 2 Institute of Pathology, Medical Faculty, University of Belgrade, 11000 Belgrade, Serbia

## Background

Adenosine is a purine nucleoside, which modifies different physiological functions, including vascular tone in numerous blood vessels. This effect is a consequence of interaction between adenosine and specific adenosine A_1_, A_2_ or A_3_ receptors. Still, the relaxant effect of this endogenous nucleoside has been shown on some blood vessels to be mainly dependent on activation of adenosine A_2_ receptors that can be located on endothelial or smooth muscle cells. To examine this assumption the aim of this study was to determine the effects of CPCA (a selective adenosine A_2_ receptor agonist) on the isolated rat femoral artery and to establish whether potassium channels are involved in this action.

## Methods

Experiments were conducted on isolated femoral arteries of male rats. Circular vascular segments were placed in an organ bath with Krebs-Ringer’s solution. Concentration-response curves for CPCA were obtained in a cumulative fashion on precontracted artery rings. Tension alterations induced by CPCA were continuously recorded.

## Results

CPCA (0.1–100 µM) produced endothelium-dependent relaxation. Incubation of DPCPX (a selective antagonist of A_1_ receptors, 10 nM) did not influence the control effect of the examined agonist, while SCH 58261 (a selective antagonist of A_2A_ receptors, 1 µM) significantly reduced CPCA-induced vasodilatation. The maximal vascular response to CPCA was comparable after denudation and incubation of SCH 58261. In the presence of high K^+^ (100 mM) a significant inhibition of the control CPCA-induced relaxation was recorded. This was not the case after the application of glibenclamide, a blocker of ATP-sensitive K^+^ channels.

## Conclusions

CPCA induced an endothelium-dependent vasodilatation of the examined blood vessel by activation of adenosine A_2A_ receptors, most probably located on the endothelial cells. It can be assumed that the CPCA-evoked action was most likely mediated via some endothelium-derived hyperpolarizing factor. However, ATP-sensitive K^+^ channels did not contribute to the overall femoral artery response to CPCA.

